# Genotyping-by-sequencing based QTL mapping for rice grain yield under reproductive stage drought stress tolerance

**DOI:** 10.1038/s41598-019-50880-z

**Published:** 2019-10-04

**Authors:** Shailesh Yadav, Nitika Sandhu, Vikas Kumar Singh, Margaret Catolos, Arvind Kumar

**Affiliations:** 1 Rice Breeding Platform, International Rice Research Institute, DAPO Box 7777, Metro Manila, Philippines; 2 Punjab Agricultural University, Ludhiana, Punjab India; 3 International Rice Research Institute, South Asia Hub, ICRISAT, Patancheru, Hyderabad India; 4 IRRI South Asia Regional Centre (ISARC), Varanasi, Uttar Pradesh 221106 India

**Keywords:** Quantitative trait, Plant breeding

## Abstract

QTLs for rice grain yield under reproductive stage drought stress (*qDTY*) identified earlier with low density markers have shown linkage drag and need to be fine mapped before their utilization in breeding programs. In this study, genotyping-by-sequencing (GBS) based high-density linkage map of rice was developed using two BC_1_F_3_ mapping populations namely Swarna*2/Dular (3929 SNPs covering 1454.68 cM) and IR11N121*2/Aus196 (1191 SNPs covering 1399.68 cM) with average marker density of 0.37 cM to 1.18 cM respectively. In total, six *qDTY* QTLs including three consistent effect QTLs were identified in Swarna*2/Dular while eight *qDTY* QTLs including two consistent effect QTLs were identified in IR11N121*2/Aus 196 mapping population. Comparative analysis revealed four stable and novel QTLs (*qDTY*_*2*.*4*_, *qDTY*_*3*.*3*_, *qDTY*_*6*.*3*_, and *qDTY*_*11*.*2*_) which explained 8.62 to 14.92% PVE. However, one of the identified stable grain yield QTL *qDTY*_*1*.*1*_ in both the populations was located nearly at the same physical position of an earlier mapped major *qDTY* QTL. Further, the effect of the identified *qDTY*_*1*.*1*_ was validated in a subset of lines derived from five mapping populations confirming robustness of *qDTY*_*1*.*1*_ across various genetic backgrounds/seasons. The study successfully identified stable grain yield QTLs free from undesirable linkages of tall plant height/early maturity utilizing high density linkage maps.

## Introduction

Genetic dissection of loci underlying drought tolerance in rice will accelerate the development of new varieties with enhanced grain yield under drought stress conditions. In this context, discovery of grain yield QTLs under drought with large and consistent effect across the genetic backgrounds and environments is the most desirable step for its successful utilization in breeding programs. Rice breeding in the last 10 years at IRRI, Philippines has witnessed the identification of robust grain yield QTLs under drought stress conditions namely, *qDTY*_*1*.*1*_^1,2^, *qDTY*_*2*.*1*_^3^, *qDTY*_*3*.*1*_^3^, *qDTY*_*12*.*1*_^4^ using SSR markers. The incorporation of such positive *qDTY* alleles has been attempted recently into high yielding rice varieties popular in rainfed lowland and rainfed upland ecosystems in Asia. Further, drought tolerant version of IR64, Swarna, Sabitri, and Sambha Mahsuri have been developed for testing and release under national/state trials in different countries^5^.

SSR markers have been successfully applied in mapping and introgression of various QTLs including *qDTYs* in rice. However, this approach is time consuming and cost ineffective due to laborious gel-based genotyping making it not feasible in the present era of available cheap DNA sequencing technologies^6^. To overcome the limitations of SSR, next generation markers such as SNPs are now available to use. SNPs are more valuable and informative markers over SSR and others due to high abundance and uniform distribution in genomes, high multiplexing and ease of automation^7^. Recent progress in next generation sequencing has developed high-throughput SNP genotyping as a rapid, precise and low-cost genotyping technique to accelerate the process of QTL mapping and gene discovery in breeding populations^8^. In rice, various fixed SNP chips *viz*., 6 K SNP chip, 44 K SNP chip, 50 K SNP chip and 700 K SNP arrays^9–12^ have been developed to find association between phenotype and genotype^13^. Further, a highly efficient and cost-effective sequence based genotyping approach called genotyping-by-sequencing (GBS) has been used for simultaneous genome wide SNP discovery and genotyping^14–16^. GBS is now the most widely used genotypic platform for crop genomics studies based on restriction enzyme digestion to reduce the complexity of genome followed by adapter ligation, PCR and sequencing^6^. Several crop species in recent years have been genotyped using this tool for various genetic studies^14,17–23^ including rice^24,25^.

GBS offers a powerful approach in producing large number of SNPs for genotyping and genetic analysis for implementing in genome wide association studies (GWAS), diversity analysis, genomic selection (GS), marker and gene discovery, genome profiling and high-resolution QTL mapping^8,26–28^. QTL mapping through GBS using high density linkage maps has been studied for various traits like fusarium wilt resistance^29^ and sterility mosaic resistance^30^ in pigeon pea, drought tolerance in chickpea^31^ rust resistance^32^ and flag leaf traits^33^ in wheat, plant architecture^23^ and yield traits^34^ in maize and grain weight, grain length in rice^35^.

In the present study, we genotyped two mapping populations segregating for grain yield using a high throughput genotyping strategy called genotyping-by-sequencing approach. The identified high-quality SNP markers were used to construct high density linkage maps in order to find consistent grain yield QTLs under drought stress situation. Further, the identified stable QTL was validated in the subset of 250 lines derived from five mapping populations.

## Results

### Phenotypic evaluation of parental lines and mapping population

Mean days to flowering (DTF), plant height (PH), grain yield (GY), heritability (H), range and least significant difference at 5% (LSD_0.05_) in four parents of two targeted mapping populations under non-stress(NS), severe stress (SS) and moderate stress (MS) conditions during 2016WS and 2017DS is presented in Table 1. The results indicated reduction in mean grain yield of parents and lines under reproductive stage drought (RS) compared with NS conditions in all the 12 experiments clearly indicating that drought stress imposed during 2016 wet season (WS) and 2017 dry season (DS) was effective. Severity of imposed drought during 2016WS was much higher than 2017DS. Drought stress delayed mean DTF in Swarna*2/Dular population by 9 days under MS while the population was accelerated by 6 days in SS compared to their mean NS trials. Mean PH was drastically reduced under both SS and MS conditions. Mean grain yield of donor parent (Dular) was reduced from 4064 kgha^−1^ (NS) to 485 kgha^−1^ and 1382 kgha^−1^ under SS and MS conditions, respectively; while that of the recipient parent (Swarna) was reduced from 4600 kgha^−1^ under NS, to 0.0 kgha^−1^ and 561 kgha^−1^ under SS and MS, respectively. The mean GY of Swarna*2/Dular population was 2894 kgha^−1^, 469 kgha^−1^ and 1382 kgha^−1^ in NS, SS and MS conditions, respectively with percent mean grain yield reduction varying from 83% in SS to 52% under MS compared to NS condition. The heritability (H) estimate ranged from 0.28 under NS to 0.30 under SS and 0.42 under MS for Swarna*2/Dular population.

**Table 1:** Mean performances for days to flowering (DTF), plant height (PH) and grain yield (GY) of two rice mapping populations (Swarna*2/Dular and IR11N121*2/Aus 196) under non-stress (NS) and reproductive stage (RS) drought conditions.

Population name	Season	Env	Stress level	DTF (Days)					PHT (cm)					GY (kgha^−1^)				
				P_1_	P_2_	M	LSD _0.05_	H	P_1_	P_2_	M	LSD _0.05_	H	P_1_	P_2_	M	LSD _0.05_	H
Swarna*2/Dular	2016WS	NS	—	105	75	92	8	0.81	112	136	139	28.5	0.8	4600	4065	2894	3570	0.28
Swarna*2/Dular	2016WS	RS	SS	96	88	86	10	0.29	70	88	87	12.0	0.46	0	485	469	735	0.30
IR11N121*2/Aus 196	2016WS	NS	—	87	86	85	5	0.51	116	129	121	14.2	0.89	4401	4314	4095	2294	0.36
IR11N121*2/Aus196	2016WS	RS	SS	86	83	83	10	0.19	96	132	116	47.6	0.55	272	589	630.6	754.6	0.49
Swarna*2/Dular	2017DS	NS	—	97	81	90	10	0.21	93	132	119	26.1	0.79	5354	4538	4740	3014	0.26
Swarna*2/Dular	2017DS	RS	MS	95	89	99	9	0.79	68	83	85	12.0	0.93	561	1382	1471	1622	0.42
IR11N121*2/Aus 196	2017DS	NS	—	85	90	86	5	0.62	93	121	104	10.9	0.92	6149	6003	5971	2593	0.39
IR11N121*2/Aus 196	2017DS	RS	MS	85	95	85	11	0.53	76	90	77	13.2	0.83	1331	1685	1787	2072	0.41

Note: Env = environment, NS = non-stress, RS = reproductive stage drought stress, P_1_ = recipient parent, P_2_ = donor parent, M = population mean, LSD_0.05_ = least significant difference at 5% confidence level, H = heritability, DTF = days to flowering in days, PHT = plant height in cm, GY (kgha^−1^) = grain yield in kg per hectare, SS = severe stress, MS = moderate stress.

Similarly, mean DTF, PH and GY, H, and LSD_0.05_ of parents and lines for IR11N121*2/Aus 196 population is also presented in Table 1. Mapping population showed mean GY 589 kgha^−1^ under SS (2016WS) and 1685 kgha^−1^ under MS (2017DS). Aus 196 (drought donor) showed 26% higher GY under MS while 116% higher GY under SS compared to IR11N121 recipient. The mean GY in IR11N121*2/Aus 196 population showed 34% and 21% higher yield than mean GY of Swarna*2/Dular population under SS (2016WS) and MS (2017DS) conditions respectively. Under NS, mean GY for IR11N121*2/Aus 196 population was also higher than GY mean of Swarna*2/Dular population in both the years during 2016 and 2017. Heritability/repeatability (H) for GY under RS was 0.49 (SS) and 0.41 (MS) while under NS ranged from 0.36 (2016WS) to 0.39 (2017DS). Mean for other yield related traits (DTF and PH) for IR11N121*2/Aus 196 population is presented in Table 1. Statistical analysis of the validation panel along with donor (Dular and Aus196) and recipient parents (TDK1, Swarna and IR11N121) is summarized in Table 2. The mean GY of validation panel was 618 kgha^−1^, 1211 kgha^−1^ and 2840 kgha^−1^ under SS, MS and NS conditions and most of the best performing lines under drought was derived from TDK 1*2/Aus196 with an average mean of 945 kgha^−1^.

**Table 2:** Trial means for DTF, PHT and GY parameters analyzed from 250 lines derived from five mapping populations (TDK1*2/Dular, TDK1*2/Aus196, Swarna*2/Dular, IR11N121*2/Aus 196, IR11N121*2/Dular) under NS and RS conditions.

Designation	DTF (days)				PHT (cm)				GY (kgha^−1^)			
	2017DS		2017WS		2017DS		2017WS		2017DS		2017WS	
	NS	MS	NS	SS	NS	MS	NS	SS	NS	MS	NS	SS
Dular	80	81	80	82	135	99	162	137	6479	2673	4069	1087
Aus196	87	89	89	93	117	87	146	105	7617	1365	4314	871
Swarna	100	112	110	117	84	65	104	86	7121	371	4500	258
TDK1	91	103	106	116	100	70	120	92	6569	500	4891	343
IR11N121	88	85	93	100	101	75	111	80	5706	1149	4743	678
Anjali	83	75	85	69	100	82	125	81	4151	748	3900	232
M	86	88	91	95	118	86	144	105	5814	1492	4228	514
LSD_0.05_	9	10	8	15	18	15	32	49	2840	1211	2446	618
H	0.87	0.89	0.85	0.84	0.84	0.79	0.39	0.42	0.54	0.56	0.37	0.57

Note: DTF = days to flowering, PHT = plant height in cm, GY (kgha^−1^) = grain yield in kg per hectare, NS = non-stress, MS = moderate stress, SS = severe stress, DS = Dry season, WS = Wet season, M = population mean, LSD_0.05_ = least significant difference at 5% confidence level, H = heritability.

### High throughput sequencing and SNP discovery

A total of 41.2 GB (307.64 million reads) and 34.8 GB (276.98 million reads) sequencing data were generated for Swarna*2/Dular and IR11N121*2/Aus 196 mapping populations, respectively (Supplementary Table S1). The sequence reads from individual lines mapped to reference genome varied from 0.23 to 11.05 million reads in Swarna*2/Dular and 0.01 to 1.31million reads for IR11N121*2/Aus 196. The sequence reads were mapped to reference genome Nipponbare IRGSP1.0^36^ (http://rapdb.dna.affrc.go.jp/) and aligned, cleaned GBS reads were used in pipeline for SNP calling. The alignment of reads to reference genome for both the populations used in this study are provided in Supplementary Tables S2 and S3). As a result, a total of 81,152 and 52,169 SNPs was identified for Swarna*2/Dular and IR11N121*2/Aus 196 mapping populations, respectively. Further, SNPs were filtered out for missing data (≤90%) and minor allele frequency (MAF) at threshold of 0.05 and in total, 6243 and 4247 high quality

SNPs were generated for both the populations to validate the allelic variations between parents and lines (Supplementary Table S4 and S5). There is a significant reduction in the numbers of SNPs from several thousands to few thousands, due to stringent selection criteria used in the present analysis. SNP calls from a nucleotide-based format were converted to parent-based format using ABH-plugin in TASSEL pipeline. After converting the generated SNPs into parent-based format, in total 3929 SNPs (Swarna*2/Dular) and 1191 SNPs (IR11N121*2/Aus 196) with contrasting alleles in parental genotype were retained to be used for construction of linkage maps.

### SNP based high density linkage maps

**Figure 1:**
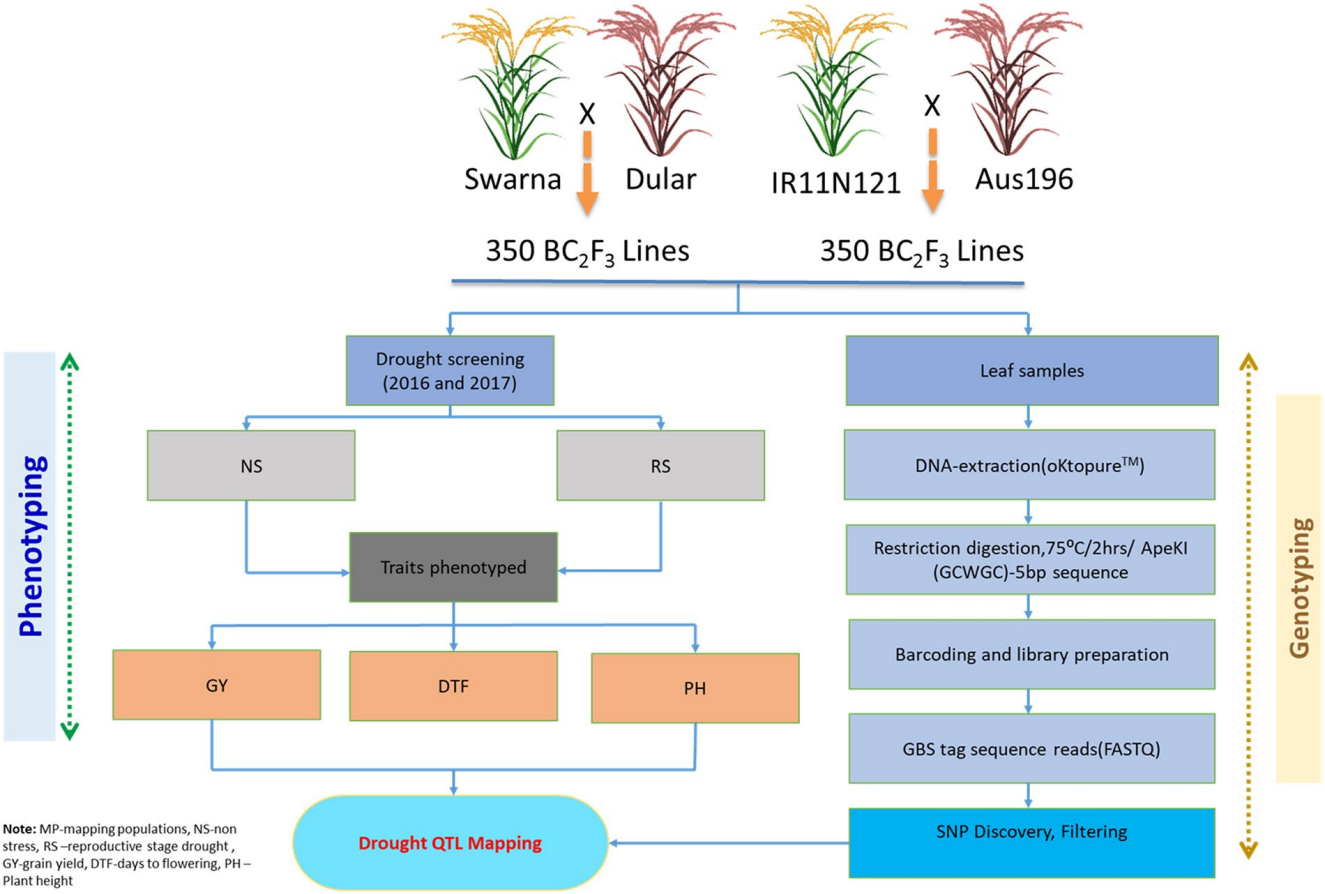
Integration of GBS derived high density SNPs and multi-season phenotyping data for mapping of drought QTLs in rice.

The genome wide polymorphic SNPs were evaluated for expected segregation ratio using Chi-square analyses in both Swarna*2/Dular and IR11N121*2/Aus 196 populations. For the Swarna*2/Dular population, a total 3929 SNPs with contrasting alleles between the parents were mapped on all 12 chromosomes while 1191 polymorphic SNPs were mapped for IR11N121*2/Aus 196 population (Supplementary Tables S6 and S7). In total, 20 SNPs for Swarna*2/Dular and 8 SNPs for IR11N121*2/Aus 196 population had shown segregation distortion and unable to locate on their respective linkage maps. The total length of genetic map computed for Swarna*2/Dular population was 1454.68 cM (varied from 89.60 cM in chromosome 9 to 169.52 cM in chromosome 1). Average genetic distance between two SNPs was 0.37 cM across the chromosomes, reflecting its utility in fine mapping of QTLs/genes. The number of SNPs mapped to each chromosome varied from 245 SNPs on chromosome 10 to 484 SNPs identified on chromosome 1 with an average of 327 SNPs per chromosome (Table 3). Similarly, genetic map of IR11N121*2/Aus 196 population consisted of 1191 SNPs with total map length of 1399.8 cM (Table 3). The number of SNPs varied from 47 SNPs on chromosome 6 to 171 SNPs on chromosome 1 with map length of 99.53 cM to 168.97 cM. An average of 99 SNPs were mapped on each chromosome for this population. A calculated average genetic distance between two SNPs across the chromosomes was 1.18 cM (ranged from 0.70 cM on chromosome 4 to 2.12 cM on chromosome 6). The developed high-density genetic maps using filtered polymorphic SNPs were integrated with data for grain yield under drought and its associated traits for QTL analysis (Fig. 1).

**Table 3:** Features of the genetic maps in Swarna*2 × Dular and IR11N121*2 × Aus 196 drought mapping populations in rice.

Swarna*2 × Dular					IR11N121*2 × Aus 196			
Chromosome Number	Filtered SNPs	SNPs mapped	Distance (cM)	Average marker distance	Filtered SNPs	SNPs mapped	Distance (cM)	Average marker distance
1	786	484	169.52	0.35	547	171	168.97	0.99
2	590	360	140.63	0.39	410	95	130.23	1.37
3	477	307	142.53	0.46	314	79	139.23	1.76
4	610	417	139.16	0.33	482	198	139.16	0.70
5	492	348	116.67	0.34	326	116	110.15	0.95
6	579	367	121.80	0.33	257	47	99.53	2.12
7	511	311	113.32	0.36	329	108	109.84	1.02
8	345	230	110.37	0.48	216	60	107.87	1.80
9	412	300	89.60	0.30	309	99	88.87	0.90
10	459	245	90.15	0.37	385	78	89.60	1.15
11	548	310	113.27	0.37	369	58	110.40	1.90
12	434	250	107.66	0.43	303	82	105.78	1.29
Total	6243	3929	1454.68	0.37	4247	1191	1399.68	1.18

### QTL analysis

#### Swarna*2/Dular population

**Figure 2:**
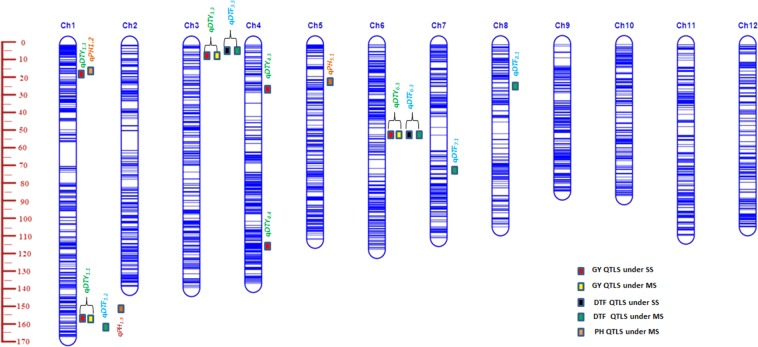
Genotyping-by-sequencing (GBS) derived high density genetic map and distribution of QTLs associated with drought tolerance in Swarna*2/Dular population. The twelve chromosomes were shown as vertical bars and each horizontal line on the bar represent single SNP marker. Aggregation on horizontal lines reflects higher marker density on that chromosome. The scale on left side represents genetic position in cM.

A total of six *qDTY* QTLs were identified for GY under severe (SS) and moderate (MS) drought conditions through composite interval mapping (CIM) during the years of 2016 and 2017. Over the two years of testing, three drought QTLs (*qDTY*_*1*.*1*_ *qDTY*_*3*.*3*_ and *qDTY*_*6*.*3*_) were detected in both SS and MS conditions during 2016WS and 2017DS while the remaining three QTLs (*qDTY*_*1*.*3*_, *qDTY*_*4*.*3*_ and *qDTY*_*4*.*4*_) were found only under SS condition during 2016WS (Table 4 and Fig. 2). These identified QTLs explained phenotypic variance (PVE) from 4.34 to 13.50% with LOD scores ranging from 2.87 to 32.05. The majority of the GY QTLs under SS and MS had positive additive effect indicating that alleles contributed from parent Dular increased the phenotypic values. Three QTLs namely *qDTY*_*1*.*1*_, *qDTY*_*3*.*3*_ and *qDTY*_*6*.*3*_ were identified as a consistent effect grain yield QTL expressed across the seasons under both SS and MS conditions of drought. The consistent effect QTL, *qDTY*_*1*.*1*_ within the marker interval of S1_40013502–S1_41216734 was positioned at 159.9–161.4 cM on chromosome 1 and explained PVE ranging from 9.45% to 10.90% with LOD scores of 3.13 to 3.89 during 2016 and 2017 respectively. The QTLs *qDTY*_*3*.*3*_ detected on chromosome 3 with PVE 13.50% and *qDTY*_*6*.*3*_ on chromosome 6 with PVE of 8.62% were novel and had consistent effect on grain yield under drought. Two QTLs *qDTY*_*3*.*3*_ and *qDTY*_*1*.*1*_ explained the highest % PVE for GY under drought from Swarna*2/Dular population.

**Table 4:** Results of QTL analysis in Swarna*2/Dular backcross mapping population in rice.

Trait	QTL Name	Stable QTLs^^^	Chrom^*^	Position^$^	Marker Interval	Previously mapped	LOD^§^	PVE (%)^¶^	Add^††^	Dom^‡‡^	Left CI^#^	Right CI^#^
GY_*MS*_	*qDTY*_*1*. *1*_	*qDTY*_*1*. *1*_	1	159.9	S1_40013502–S1_40089754	36.75–40.70 Mb^1^	3.89	10.90	141.56	−256.0	156.4	160.4
GY_*SS*_	*qDTY*_*1*. *1*_		1	161.4	S1_41176753–S1_41216734		3.13	9.45	135.35	−211.46	161.2	161.5
GY__*MS*_	*qDTY*_*3*. *3*_	*qDTY*_*3*. *3*_	3	10.2	S3_2625614–S3_2686581	novel QTL	4.18	11.42	231.89	−177.33	10.1	10.7
GY__*SS*_	*qDTY*_*3*. *3*_		3	10.4	S3_2686581–S3_2727277		7.80	13.50	1.22	1285.29	10.4	10.9
GY__*SS*_	*qDTY*_*6*. *3*_	*qDTY*_*6*. *3*_	6	57.8	S6_14604291–S6_15072250	novel QTL	21.26	4.91	6.36	1078.65	57.3	58.3
GY__*MS*_	*qDTY*_*6*. *3*_		6	58.8	S6_14604291–S6_15072250		2.98	8.62	16.45	447.55	57.3	59.3
GY__*SS*_	*qDTY*_*1*. *3*_	—	1	21.9	S1_5575869–S1_5622569	—	7.17	4.52	2.79	832.30	21.4	22.4
GY__*SS*_	*qDTY*_*4*. *3*_	—	4	29.2	S4_7142266–S4_8718094		32.05	4.90	7.32	1102.25	28.7	29.7
GY__*SS*_	*qDTY*_*4*. *4*_	—	4	119.2	S4_30374971–S4_30570019	—	2.87	4.34	−5.85	371.073	118.7	119.7
DTF__*MS*_	*qDTF*_*3*. *3*_	*qDTF*_*3*. *3*_	3	8.4	S3_1990671S3_2352329	—	3.68	5.96	−2.19	−0.96	6.9	8.9
DTF__*SS*_	*qDTF*_*3*. *3*_		3	9.6	S3_2467421S3_2625614		4.25	8.53	−7.19	−0.58	9.6	10.4
DTF__*SS*_	*qDTF*_*6*. *3*_	*qDTF*_*6*. *3*_	6	57.8	S6_14604291S6_15072250	—	5.26	8.15	−6.58	44.73	57.1	58.7
DTF__*MS*_	*qDTF*_*6*. *3*_		6	58.8	S6_14604291S6_15072250		3.67	7.74	1.52	−3.13	58.3	59.3
DTF__*MS*_	*qDTF*_*1*. *2*_	—	1	167.9	S1_42655097S1_42885648	—	3.00	4.95	−1.78	0.4	167.4	168.9
DTF__*MS*_	*qDTF*_*7*. *1*_	—	7	75.1	S7_18706568S7_19334027	—	6.48	4.78	−3.07	2.46	74.6	75.6
DTF__*MS*_	*qDTF*_*8*. *1*_	—	8	27.1	S8_6585662S8_7225748	—	6.14	9.72	0.04	24.42	25.6	27.6
PH__*MS*_	*qPH*_*1*. *2*_	—	1	17.9	S1_4486055S1_4950915	—	4.08	5.53	−3.75	−0.91	17.4	18.4
PH__*MS*_	*qPH*_*1*. *3*_	—	1	150.9	S1_38286810S1_38613195	—	25.79	27.66	8.80	5.33	150.4	151.4
PH__*MS*_	*qPH*_*5*. *1*_	—	5	26.5	S5_6678640S5_6883481	—	3.36	3.52	3.38	−3.12	26.2	27.1

Note: ^^^QTLs detected in both the years (2016 and 2017) under SS and MS conditions of drought. ^*^Chromosome number on which QTL was identified. ^$^The scanning position in cM on the chromosome. ^§^LOD score calculated from composite interval mapping. ^¶^Phenotypic variation explained by QTL. ^††^Estimated additive effect of QTL. ^‡‡^Dom: Estimated dominance effect of QTL. ^#^Confidence interval calculated by one-LOD drop from the estimated QTL position, DTF = days to flowering in days, PH = plant height in cm, GY (kgha^−1^) = grain yield in kg per hectare.

Five QTLs for days to flowering (DTF) including two stable ones were expressed under both SS and MS conditions while three QTLs for PH were identified in MS conditions only (Table 4 and Fig. 2). Two significant QTLs (*qDTF*_*3*.*3*_ and *qDTF*_*6*.*3*_) for DTF mapped under both SS and MS conditions were consistent and one of them (*qDTF*_*6*.*3*_) was located at similar genetic locations with the grain yield QTL *qDTY*_*6*.*3*_ in Swarna*2/Dular population. One of the DTF QTL (*qDTF*_*1*.*1*_) detected under SS in marker interval of S1_42655097S1_42885648 was detected nearly 1 Mb away from the GY QTL under drought, *qDTY*_*1*.*1*_ identified in Swarna*2/Dular population. Phenotypic variations explained by DTF QTLs were significantly low (4 to 6%) as compared to GY QTLs under drought (4.34 to 13.50%). For plant height (PH), three QTLs (*qPH*_*1*.*2*_, *qPH*_*1*.*3*_ and *qPH*_*5*.*1*_) were detected with PVE ranged from 3 to 27% under MS on chromosomes 1 and 5. No stable QTL for PH under drought had been identified in Swarna*2/Dular population.

#### IR11N121*2/Aus 196 population

**Figure 3:**
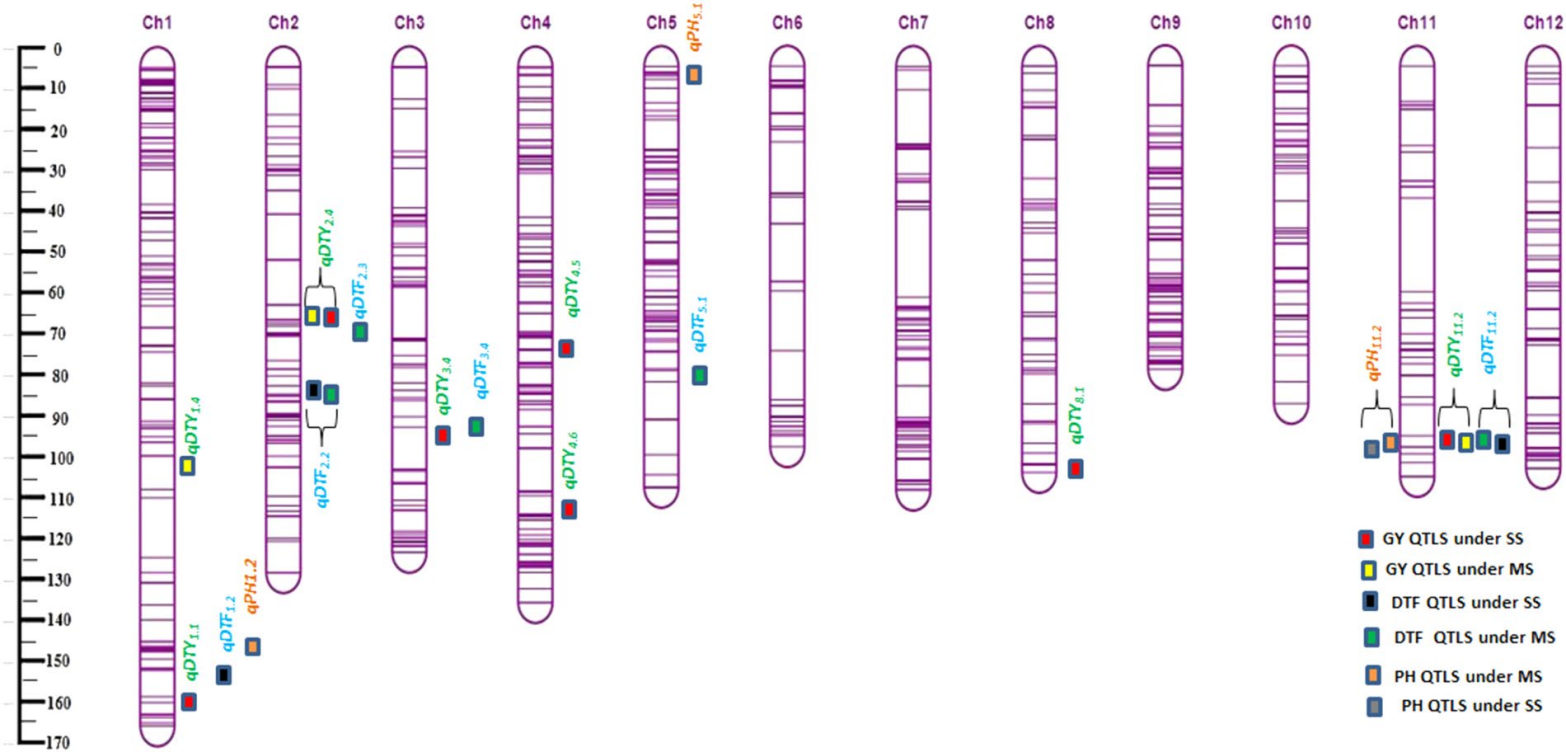
Genotyping by sequencing (GBS) derived high density genetic map and distribution of QTLs associated with drought tolerance in IR11N121*2/Aus 196 population. The twelve chromosomes were shown as vertical bars and each horizontal line on the bar represent single SNP marker. Aggregation on horizontal lines reflects higher marker density on that chromosome. The scale on left side represents genetic position in cM.

A total of eight *qDTY* QTLs for GY under severe (SS) and moderate (MS) drought conditions were detected from IR11N121*2/Aus 196 population during the years of 2016WS and 2017DS (Table 5 and Fig. 3. These QTLs explained from 4.56 to 14.92% of the PVE and were distributed over the six chromosomes (1, 2, 3, 4, 8 and 11). Four QTLs (*qDTY*_*1*.*1*_, *qDTY*_*3*.*4*_, *qDTY*_*4*.*5*_ and *qDTY*_*4*.*6*_) were detected under SS in 2016 and two QTLs (*qDTY*_*1*.*4*_ and *qDTY*_*8*.*1*_) identified under MS in 2017. Two QTLs (*qDTY*_*2*.*4*_ and *qDTY*_*11*.*2*_) was found as a stable QTLs identified in both the environments (MS and SS) during 2016 and 2017. The stable QTL *qDTY*_*2*.*4*_ in marker interval of S2_16924409–S2_17554671 had explained PVE ranged from 8.84 to 14.92% while *qDTY*_*11*.*2*_ was flanked by markers S11_23405441 and S11_25462601 with PVE ranged from 4.75 to 9.35% under MS and SS conditions respectively. Additive effect for most of the QTLs except *qDTY*_*1*.*4*_, *qDTY*_*3*.*4*_, *qDTY*_*8*.*1*_ had negative values suggesting parent IR11N121 contributed alleles for increased GY under drought. The QTL *qDTY*_*1*.*1*_ identified under MS in this population (IR11N121*2/Aus 196) was also detected in Swarna*2/Dular population under MS and SS drought conditions. This common QTL *qDTY*_*1*.*1*_ in both the populations lying in the QTL-region of *qDTY*_*1*.*1*_, may have a key region of rice genome to explore the underlying variation related to drought. Several QTLs for DTF and PH under SS and MS conditions were observed at chromosomes 1, 2, 3, 5 and 11 under SS or MS conditions (Table 5 and Fig. 3). However only two of the identified QTLs (*qDTF*_*2*.*2*_ and *qDTF*_*11*.*2*_) were detected in both SS and MS conditions across the years. Stable QTLs for DTF namely, *qDTF*_*2*.*2*_ and *qDTF*_*11*.*2*_ had explained PVE from 3 to 11%. The positive allele for duration increase was contributed by IR11N121 parent for most of the DTF QTLs except *qDTF*_*3*.*4*._ One of the DTF QTL *qDTF*_*11*.*2*_ was co-located with *qDTY*_*11*.*2*_ detected in IR11N121*2/Aus 196 population. Four QTLs for PH including one stable QTL (*qPH*_*11*.*2*_) were mapped at chromosomes 1, 5 and 11. The plant height QTL *qPH*_*11*.*1*_ was also co-located with QTL for DTF (*qDTF*_*11*.*2*_) and GY (*qDTY*_*11*.*2*_) under drought in IR11N121*2/Aus 196 population.

**Table 5:** Results of QTL analysis in IR11N121*2/AUS 196 backcross mapping population in rice.

Trait	QTL Name	Stable QTLs^^^	Chrom^*^	Position^$^	Marker Interval	Previously mapped	LOD^§^	PVE (%)^¶^	Add^††^	Dom^‡‡^	Left CI^#^	Right CI^#^
GY__*SS*_	*qDTY*_*2*. *4*_	*qDTY*_*2*. *4*_	2	69.4	S2_17630922–S2_17731936	novel QTL	2.92	14.92	−86.62	−511.43	68.9	69.9
GY__*MS*_	*qDTY*_*2*. *4*_		2	67.4	S2_16924409–S2_17554671		6.81	8.84	−14.39	1361.98	66.3	68.1
GY__*SS*_	*qDTY*_*11*. *2*_	*qDTY*_*11*. *2*_	11	98.1	S11_25462601–S11_26923782	novel QTL	7.35	9.35	−923.53	−927.91	95.6	98.6
GY__*MS*_	*qDTY*_*11*. *2*_		11	105.1	S11_27252113–S11_28165211		2.75	4.75	133.04	243.107	105.6	108.6
GY*_*_*SS*_	*qDTY*_*1*. *1*_	—	1	163.6	S1_41767801–S1_42906879	36.75–40.70 Mb^1^	2.95	5.55	−182.08	−4.60	163.1	168.6
GY__*MS*_	*qDTY*_*1*. *4*_	—	1	107.6	S1_25580728–S1_27768807	—	2.82	4.82	125.042	455.64	104.1	109.1
GY__*SS*_	*qDTY*_*3*. *4*_	—	3	93.4	S3_23410049–S3_24443082		2.52	4.52	42.36	1009.02	92.9	95.9
GY__*SS*_	*qDTY*_*4*. *5*_	—	4	76.8	S4_18967234–S4_19812844		2.98	4.98	−48.57	1135.91	76.3	77.3
GY__*SS*_	*qDTY*_*4*. *6*_	—	4	116.8	S4_29797214–S4_29868104	—	3.05	5.05	−127.12	78.13	116.3	117.3
GY__*MS*_	*qDTY*_*8*. *1*_	—	8	107.6	S11_27252113–S11_28165211	24–26 Mb^65^	2.96	4.56	202.67	270.53	106.1	107.6
DTF__SS_	*qDTF*_*2*. *2*_	*qDTF*_*2*. *2*_	2	86.4	S2_22001414–S2_22831782	—	13.29	3.21	−0.31	−80.67	85.98	86.98
DTF__MS_	*qDTF*_*2*. *2*_		2	90.4	S2_23011317–S2_23246520	—	12.96	3.29	−0.72	−80.90	89.98	90.98
DTF__MS_	*qDTF*_*11*. *2*_	*qDTF*_*11*. *2*_	11	97.1	S11_23405441–S11_25462601	—	4.68	11.10	−4.53	−4.40	95.62	98.62
DTF__SS_	*qDTF*_*11*. *2*_		11	100.1	S11_25462601–S11_26203565	—	13.13	3.21	40.26	40.25	99.62	100.6
DTF__SS_	*qDTF*_*1*. *1*_		1	164.6	S1_39508386–S1_41216734	—	15.00	5.21	−40.44	39.68	163.1	166.1
DTF__MS_	*qDTF*_*2*. *3*_		2	70.4	S2_17732007–S2_19367035	—	4.47	3.08	−16.57	17.09	69.98	70.98
DTF__MS_	*qDTF*_*3*. *4*_		3	97.4	S3_24443082–S3_24882499	—	4.68	5.16	0.83	−0.58	95.91	97.91
DTF__MS_	*qDTF*_*5*. *1*_		5	83.3	S5_21166454–S5_23610966	—	11.07	10.03	−8.29	−8.43	82.83	83.83
PH__MS_	*qPH*_*11*. *2*_	*qPH*_*11*. *2*_	11	98.1	S11_23405441–S11_25462601	—	4.72	6.06	−8.31	−8.87	96.62	99.62
PH__SS_	*qPH*_*11*. *2*_		11	104.1	S11_26203632–S11_26665891	—	5.05	5.30	46.01	49.62	103.6	104.6
PH__MS_	*qPH*_*1*. *1*_		1	152.6	S1_38752441–S1_39380942	—	6.90	7.71	−5.53	−1.47	152.1	153.1
PH__MS_	*qPH*_*5*. *1*_		5	4.3	S5_951816–S5_1195956	—	3.11	1.82	3.25	1.43	3.83	5.83

Note: ^^^QTLs detected in both the years (2016 and 2017) under SS and MS conditions of drought. ^*^Chromosome number on which QTL was identified. ^$^The scanning position in cM on the chromosome, ^§^LOD score calculated from composite interval mapping, ^¶^Phenotypic variation explained by QTL. ^††^Estimated additive effect of QTL, ^‡‡^Dom: Estimated dominance effect of QTL, ^#^Confidence interval calculated by one-LOD drop from the estimated QTL position, DTF = days to flowering in days, PH = plant height in cm, GY (kgha^−1^) = grain yield in kg per hectare.

### Co-localization of *qDTY* QTLs

Many of the previously reported *qDTY* QTLs including *qDTY*_*1*.*1*_ were linked with undesirable alleles of tallness/earliness and fine mapping approach was to be followed before introgression of any such QTLs. In the present study, we have detected four stable QTLs (*qDTY*_*2*.*4*_, *qDTY*_*3*.*3*_, *qDTY*_*6*.*3*_ and *qDTY*_*11*.*2*_)_._ One QTL (*qDTY*_*1*.*1*_) was co-located at the same position as previously reported. We found that *qDTY*_*6*.*3*_ was linked with the QTL for DTF (*qDTF*_*6*.*3*_) and *qDTY*_*11*.*2*_ with the QTLs for DTF (*qDTF*_*11*.*2*_) and PH (*qPH*_*11*.*2*_).

### Validation of *qDTY*_*1*.*1*_ allele across the five mapping populations

We identified a common grain yield QTL (*qDTY*_*1*.*1*_) between 40.01 to 41.21 Mb (1.2 Mb) expressed in both the mapping populations (Swarna*2/Dular and IR11N121*2/Aus 196) under RS drought conditions and it was overlapped with the chromosomal regions carrying *qDTY* QTLs detected within 36.75–40.70 Mb earlier by Vikram *et al*.^1^. The SSR markers present within the vicinity of the newly mapped QTL region was utilized on 250 lines derived from five mapping populations to validate the *qDTY*_*1*.*1*_. Out of tested six SSRs, two markers (RM12091and RM12146-at 40.21 Mb & 40.7 Mb respectively) were found polymorphic between the parents and were utilized for the validation. SSR markers reported in the study, presented in the vicinity of the fine mapped QTLs (1.2 Mb), free from undesirable linkages shall be utilized in the breeding programs. Lines with and without *qDTY*_*1*.*1*_ QTL and their GY data under drought is provided in Table S8. A significant difference was found in comparative mean for grain yield under drought among the lines with having *qDTY*_*1*.*1*_ and lines without having *qDTY*_*1*.*1*_ QTL (Table S9). The estimation of QTL effect had shown an advantage of 473.43 kgha^−1^ in the lines with having positive allele for *qDTY*_*1*.*1*_ QTL compared to the lines without *qDTY*_*1*.*1*_ QTL (Table S9).

### Candidate genomic regions for breeding for drought tolerance

In this study, three novel *qDTYs* (*qDTY*_*2*.*4*_, *qDTY*_*3*.*3*_ and *qDTY*_*6*.*3*_) constitutively expressed under variable situations of drought from moderate to severe conditions were found consistent on grain yield under drought with the maximum variation of 14.92% explained by *qDTY*_*2*.*4*_. The QTL size for above mentioned QTLs was varied from 0.1–1.0 Mb and free from undesirable linkages to plant height. One of the stable QTL (*qDTY*_*1*.*1*_) detected between 40.01 to 41.21 Mb (1.2 Mb) in this study was located near to the previously mapped *qDTY*_*1*.*1*_ within 36.75–40.70 Mb on the long arm of chromosome 1. The undesirable linkages such as tallness and earliness in maturity started from 36.70–38.89 Mb has been linked with drought QTL (*qDTY*_*1*.*1*_) mapped upto 40.70 Mb in the previous study by Vikram *et al*.^1^. The *qDTY*_*1*.*1*_ with detected in this study was free from undesirable linkages due to minimal QTL size of 1.2 Mb compared to 3.95 Mb QTL size of previous mapped by Vikram *et al*.^1^. A comparative map showing the narrowed down genomic region of previously mapped *qDTY*_*1*.*1*_ using high density linkage map in the present study has been depicted in Supplementary Fig. S1(a,b). The genomic regions underlying these fine mapped QTLs (*qDTY*_*2*.*4*_, *qDTY*_*3*.*3*_ and *qDTY*_*6*.*3*_) including *qDTY*_*1*.*1*_ will be an important candidate region for its utilization in marker assisted selection, sequencing and allele mining for drought tolerance in rice.

## Discussion

Marker assisted breeding had enormous potential to achieve desirable phenotypic variation in less time through deployment of markers linked to QTLs for desirable trait^37^. However, discovery and development of SSR markers, their scoring across populations is time consuming, labor intensive and costly process^38,39^. Even large QTL regions identified through low density SSR markers may introduce undesirable linkages through MAS and can make the introgression line unacceptable for release and cultivation^40^. Such limitations of SSR marker makes it unrealistic in fine mapping of complex traits and for full use in accelerated breeding programme compare to recent available sequencing technologies at much cheaper cost. In some of the recent studies, undesirable linkages were successfully eliminated using next generation markers such as SNPs by identifying the recombinants to break linkage a favorable allele conferring drought tolerance and an unfavorable allele for tall plant height^41^. Currently, next generation sequencing (NGS) technologies have become powerful tools for discovery of millions of SNPs in cost effective manner to develop high density linkage maps, dissect the complex traits and identify key genomic regions underlying the associated traits.

A rapid, robust and cost-effective genotypic platform called GBS is nowadays becoming more feasible in genomics assisted breeding by providing many markers for QTL/gene discovery at much cheaper rate^14,18,42^. In this study, we have developed two high density genetic maps using GBS approach to identify QTLs for grain yield under various drought conditions (SS and MS) using multi-season phenotypic data from two mapping populations. The average inter-marker distance between two SNPs varied from 0.37 cM to 1.18 cM in both populations used in this study which may be one of the most saturated genetic maps developed for QTL identification in rice for drought tolerance. Drought donors (Dular and Aus 196) used in this study for population development belongs to a distinct genetic group so called *aus*-type^43^ and known for valuable genetic resource for abiotic stress tolerance including drought^5,44^. Recently, drought-responsive metabolite associated with tolerance had been identified in two *Aus* rice varieties (Dular and N22) underlying genes and pathways for drought tolerance in rice^45^. Both donors (Dular and Aus 196) used in this study gave significantly higher yields than the recipient parents (Swarna, IR11N121 and TDK1) under various drought conditions tested over two years, suggesting their usefulness and reliability in utilization as donors.

Drought screening in rain-out shelter during 2016WS was more effective than field screening in 2017 DS and could be attributed to precise control and monitoring of the amount and timing of irrigation. Broad-sense heritability (H) for GY was moderate under SS, MS and NS conditions while it was moderate to high for DTF and PH in both the populations and in both years. Previous studies also reported moderate heritability of grain yield under drought and high heritability for DTF and PH under non-stress and drought conditions^5,46,47^.

A total of fourteen QTLs for GY under SS and MS conditions were detected from the two populations used in this study. Most of the GY QTLs identified in this study were expressed either in SS or MS conditions of drought and only few of them were detected in both SS and MS drought across the years 2016 and 2017. Three such QTLs (*qDTY*_*1*.*1*_, *qDTY*_*3*.*3*_ and *qDTY*_*6*.*3*_) for Swarna*2/Dular and two QTLs (*qDTY*_*2*.*4*_ and *qDTY*_*11*.*2*_) in IR11N121*2/Aus 196 population were found stable across the environments/seasons. The lack of stability of QTL effects across the environments/genetic backgrounds has been one of the most limiting factors in successful deployment of QTLs through MAS breeding for various complex traits including drought^5,48–50^.

One of the stable grain yield QTL *qDTY*_*3*.*3*_ identified in our study was located far away from previously mapped *qDTY*_*3*.*1*_ at 30–31 Mb physical position in rice genome reported by Venuprasad *et al*.^3^. One of the GY QTLs *qDTY*_*4*.*3*_ identified at 0.7–0.8 Mb in Swarna*2/Dular population under SS was co-located with *qDTY*_*4*.*1*_ reported earlier by Swamy *et al*.^51^, however this QTL was not detected under moderate level of drought stress in the present study. It is interesting to note that we have detected QTL *qDTY*_*1*.*1*_ with significant effects under varying severity of drought (SS and MS) in both the populations used in this study. Also, the physical position (S1_40013502–S1_41216734) of this QTL was nearly same of previously identified QTL *qDTY*_*1*.*1*_^1^ a most relevant grain yield QTL under drought. It indicates authenticity, reliability of this study and usefulness of the stable QTLs under drought.

Furthermore, we have validated the effect of this QTL *qDTY*_*1*.*1*_ in five alternate mapping populations derived from three genetic backgrounds (TDK1, Swarna and IR11N121) developed in this study. Consistency of *qDTY*_*1*.*1*_ in multiple genetic backgrounds were also found in many previous studies in both (lowland and upland) the ecosystems of rice^1,2,52^. The positive alleles of *qDTY*_*1*.*1*_ was found in 64%^53^ and >50%^54^ of the drought tolerant lines from a panel of random drought-tolerant lines used. These findings clearly suggest that the *qDTY*_*1*.*1*_ on chromosome 1 could be a hot spot for alleles with positive effect on GY under drought and useful candidate region for explore in genomics assisted breeding.

Two of the consistent QTL *qDTY*_*2*.*4*_ and *qDTY*_*11*.*2*_ in IR11N121*2/Aus 196 population located at 17 Mb and 27 Mb were far from *qDTY*_*2*.*1*_^3^ and *qDTY*_*11*.*1*_^55^. The QTLs for GY under drought with PVE upto 14.92% were detected in this study, which was low, despite using the highly saturated genetic map and multiple season of precise phenotyping. However, use of high-density maps could be helpful in precise detection of QTLs for complex traits by reducing the chances of getting false positive^29^. Similar finding was discussed in earlier reports, where upto 15% PVE was achieved using GBS based QTL mapping for fusarium wilt resistance in pigeon pea and flag leaf traits in bread wheat^29,33^. Most of previously identified major effect drought QTLs except *qDTY*_*12*.*1*_ had explained 10 to 30% PVE with yield advantage of 300–500 kg ha^−1^ under RS drought stress^5,56^ and QTL pyramiding looks a feasible strategy here to achieve the desired level of phenotypic variation (yield advantage of 1.0 t ha^−1^) under severe drought stress^5,57^.

In this study, a stable GY QTL stable *qDTY*_*6*.*3*_ was co-located with QTLs for DTF *qDTF*_*6*.*3*_ in Swarna*2/Dular population while *qDTY*_*11*.*2*_ was co-located with *qDTF*_*11*.*2*_ and *qPH*_*11*.*2*_ in IR11N121*2/Aus 196 population. Many of the previous studies also reported the co-existence of drought grain yield QTLs with QTLs for DTF and PH under stress. For instance, *qDTY*_*1*.*1*_ was coinciding with QTLs for DTF and PH^1^, *qDTY*_*3*.*1*_ co-located with DTF^3^ and *qDTY*_*12*.*1*_ with QTLs for DTF, PH and other morphological traits^4^. Three QTLs (*qDTY*_*1*.*1*_, *qDTY*_*2*.*4*_, *qDTY*_*3*.*3*_) found in this study were critical and desirable loci without any linkage drag and can be introgressed in multiple genetic backgrounds to find their individual and combined effects. Transfer of major grain yield QTLs under drought co-located with PH is not preferable in MAS breeding as positive alleles of tallness could make the introgression line unacceptable for varietal release. Most of the consistent QTLs for grain yield under drought detected in this study (*qDTY*_*1*.*1*_, *qDTY*_*2*.*4*_ and *qDTY*_*3*.*3*_) were free from undesirable linkages with positive alleles of tallness/earliness except *qDTY*_*11*.*2*_ linked with QTLs for PH and DTF and *qDTY*_*6*.*3*_ had linked with QTLs for DTF. Introgression of GY QTLs unlinked from PH QTLs will led to development of rice varieties tolerant to drought with optimal plant stature and higher yield. The association of QTL for DTF and PH with grain yield QTLs under drought prevailed for two *qDTYs* found in this study and a suitable breeding strategy should be followed before introgression of such QTLs in elite varieties of rice. Recently, marker assisted linkage –elimination strategy was followed to remove the undesirable linkages of PH QTLs from grain yield QTLs such as *qDTY*_*1*.*1*_, *qDTY*_*3*.*1*_ and *qDTY*_*12*.*1*_^58^.

## Conclusion

The developed high-density genetic maps in this study could be a strong foundation for fine mapping of grain yield QTLs under drought stress and identified genomics regions could be utilized in breeding programs. We have identified some novel candidate genomic regions from two populations that contained four stable QTLs for grain yield under drought in multiple environments. Two novel *qDTY* QTLs (*qDTY*_*2*.*4*_ and *qDTY*_*3*.*3*_) along with *qDTY*_*1*.*1*_ detected at same position of previously known drought QTL *qDTY*_*1*.*1*_ in rice genome, free from any undesirable linkages have suggested the importance and utility of these QTL cluster regions for rice breeders to be utilized in MAS work and candidate gene identification for higher grain yield under drought stress situation.

## Methods

### Plant materials

Two drought tolerant donors (Dular and Aus196) and three recipient parents (Swarna, IR11N121, and TDK1), were utilized for the development of five BC_1_F_3_ bi-parental mapping populations (Swarna*2/Dular, IR11N121*2/Dular, TDK1*2/Dular, IR11N121*2/Aus 196 and TDK1*2/Aus196). Dular, a drought-tolerant donor identified at IRRI is an early maturing, low yielding, blast resistant, traditional cultivar originated from India^43^. Aus 196, an improved drought tolerant cultivar belongs to *aus* subspecies and originated from Eastern India^43,59^. Recipient parent, Swarna is a long duration, high yielding, mega variety for rainfed and irrigated rice ecosystems of India, Nepal and Bangladesh but highly susceptible to reproductive stage (RS) drought stress^1,3^. IR11N121 is a high yielding rice variety released for lowland rice ecology of South East Asia, susceptible to drought. TDK1 is a high yielding, long duration, glutinous Lao variety, highly susceptible to drought and submergence^60^.

### Phenotypic evaluation and statistical analysis

A total of 12 experiments were conducted using five mapping populations during 2016 and 2017 under non-stress (NS) and reproductive stage drought (RS) conditions at IRRI Los Baños, Laguna, Philippines (14°30′N, 121°15′E). Two BC_1_F_3_ mapping populations (Swarna*2/Dular and IR11N121*2/Aus 196) consisted of 350 lines each were used for phenotyping and genotyping in the process of QTL identification. A validation panel consisted of 250 lines randomly selected from five mapping populations [TDK1*2/Dular (50 lines), TDK1*2/Aus196 (50 lines), Swarna*2/Dular (50 lines), IR11N121*2/Aus196 (50 lines), IR11N121*2/Dular (50 lines)] was utilized for the confirmation of positive alleles of any stable QTLs found in the present study.

The two mapping populations (Swarna*2/Dular and IR11N121*2/Aus 196) were screened under RS and NS conditions during 2016 and 2017. The 2016 wet season (WS) reproductive stage drought stress phenotyping screening was performed at IRRI rain out shelter facility while 2017 dry season (DS) screening was carried out directly in field. Experiments were laid out in augmented- RCBD design using repeated drought tolerant (Sahbhagi dhan) and susceptible checks (Swarna, IR64 and MTU1010), along with parents in 5-meter row plot with row spacing of 0.20 m under both NS and RS conditions. A total of 250 lines pooled from five mapping populations as described above for QTL validation were also phenotyped under NS and RS in alpha-lattice design with 2 replications during 2017DS in field and 2017WS in rain out shelter.

Crop management practices in the field were followed as in Vikram *et al*.^1^. The standard protocol for reproductive stage drought (RS) screening was adopted as described previously by Kumar *et al*.^5^. In brief, the stress was imposed by draining out water from the field at 50 DAS (days after seeding) in 2016WS and 2017DS, respectively and the cyclic soil moisture deficit stress was maintained till the maturity stage. Water table depth was measured using PVC pipe of 1.1 m length installed at regular places across the field. When water table level in the PVC fell below 1 meter from soil surface and all the susceptible checks started showing severe leaf rolling and dying, a life-saving irrigation through flash flooding was provided. Water was drained out immediately after 24 hrs to initiate next cycle of stress.

Data on days to 50% flowering (DTF) in days, plant height (PH) in cm and grain yield (GY) in kg ha^−1^ (GYKGPHA) from NS and RS trials were collected and analyzed using PBTools (http://bbi.irri.org/products) for computation of means, LSD and heritability (H). Experiments with grain yield reduction of more than 65% were classified as severe stress (SS), while 31–64% yield reduction was classified as moderate stress (MS)^39^. Linear mixed model was used for analysis of variance considering the lines/genotypes as fixed and the effect of replications and blocks within replications as random.

The model used for augmented-RCBD design was:

$$Yijk=M+Gi+Bl+Eilk$$

where, Yijk is measurement recorded in plot, M is the overall mean of plot, Gi is the effect of the i^th^ genotype, Bl is the effect of the l^th^ block and E_ilk_ is the experimental error.

The model used for alpha-lattice design was:

$$Yijk=M+Gi+Rj+Blj+Eilk$$

where, Yijk is measurement recorded in plot, M is the overall mean of plot, G_i_ is the effect of the i^th^ genotype, Rj is the effect of the jth replicate, Blj is the effect of the l^th^ block within jth replicate, and *E*_*ilk*_ is the experimental error.

To estimate broad sense heritability (H), variance components were estimated for a model using PBTools software packages by considering all the variables and genotypes as random. Broad sense heritability (H) or repeatability was calculated as

$${\rm{H}}={{\rm{\sigma }}}_{{\rm{G}}}^{2}/({{\rm{\sigma }}}_{{\rm{G}}}^{2}+{{\rm{\sigma }}}_{{\rm{E}}}^{2}/{\rm{r}})$$

where, H is broad sense heritability, ${{\rm{\sigma }}}_{{\rm{G}}}^{2}$ represents the genetic variance, ${{\rm{\sigma }}}_{{\rm{E}}}^{2}$ the error variance and r the number of replications.

The QTL effect on grain yield under drought stress were estimated using mixed model analysis (REML) in CROPSTAT version 7.2.3 using the lines with and without QTL. The effects of the QTL and genotypes within the QTL are considered as fixed while the replicate and blocks within replicate effects are considered random. We first computed the genotype means using PB tools by adjusting blocks in augmented RCBD design and in second stage these genotype means were used to analyze the QTL mean comparisons involving the two classes “with” and “without” QTLs using CROPSTAT.

### Leaf tissue sampling, DNA extraction and preparation of GBS libraries

The fresh leaf tissue samples from six plants per line were collected at 42 DAS. High throughput automated leaf sampling and genomic DNA extraction from leaf tissue was performed using Brooks PlantTrak Hx rice leaf tissue sampler and LGC Genomics oKtopure systems at IRRI genotyping service laboratory. The assessment of DNA quality and quality was done by running on 1% agarose gel. Using this platform, a high-quality DNA yield ranges from 40–60 ng/µl was achieved for SNP genotyping. GBS libraries were prepared using the protocol adapted from Elshire *et al*.^14^. For GBS a type II restriction endonuclease (*ApeKI*) was used for DNA digestion, and the digested DNAs were ligated to the adapter, and then 96-plex library was constructed as per GBS protocol^14^. GBS was carried out using HiSeq2500 sequencing platform with Macrogen Inc. (Korea).

### SNP identification and genotyping

The sequence reads generated in FASTQ file were processed and analyzed for SNP identification using TASSELGBS analysis pipeline^61^. Pipeline allows searching of all raw sequencing reads with perfectly matched barcode and expected remnant bp of restriction cut site and reads were further sorted, de-multiplexed and trimmed to create a unique, 64-bp long sequences called tags. These good quality tag sequences were aligned with the reference genome using Burrows-Wheeler Alignment (BWA) software^62^, while reads carrying “N” within first 64 bases had removed from further analysis. The perfectly matched and aligned sequences was processed further for SNP calling and genotyping through GBS analysis pipeline. SNPs were further filtered for minor allele frequency (MAF) below 0.05 and for the percentage of missing data (≤90%) per SNP using TASSEL GBS analysis pipeline using default parameters. Filtered data file having final set of SNPs in nucleotide-based hap map format was converted to an ABH-based format using ABH-plugin in TASSEL pipeline where “A” represents donor allele, “B” represents recipient allele and “H” represents heterozygous allele. Finally, imputed SNPs of lines were filtered against parental alleles and only polymorphic SNPs were retained to be used in construction of linkage map.

### Linkage map construction and QTL mapping

The genotypic data for 350 lines from each mapping population with filtered SNP markers was used for linkage map construction using the linkage mapping function implemented in the QTL IciMapping software v4.1^63^. The grouping and ordering of 3929 and 1191 polymorphic SNP markers for both the populations were carried out using regression mapping algorithm RECORD (REcombination Counting and ORDering) based on recombination events between adjacent markers. Further, Rippling was done for fine-tuning of the ordered markers on their respective chromosomes by sum of adjacent recombination fractions (SARF) algorithm with a default window size. QTL mapping for GY QTLs and other traits under drought was performed using composite interval mapping (CIM) functions implemented in the QTL IciMapping software v4.1^64^. The threshold LOD value to declare a significant.

QTL was computed by a permutation test involving 1000 runs at a significance level of p = 0.05. After completion of permutation test, window size of 10 cM and walk speed of 1 cM was set to start analysis of composite interval mapping.

## Supplementary information

Supplementary Information

Table S1

Table S2

Table S3

Table S4

Table S5

Table S6

Table S7

Table S8

Table S9

## Data Availability

The data sets supporting the results of this article are included within the article.
