# Supplementary material for: Genotyping-by-sequencing based QTL mapping for rice grain yield under reproductive stage drought stress tolerance
Source: Sci Rep. 2019 Oct 4;9:14326. doi: 10.1038/s41598-019-50880-z (PMC6778106; doi:10.1038/s41598-019-50880-z)
Supplement: Supplementary file 1 — Supplementary Information [file 41598_2019_50880_MOESM1_ESM.pdf]

Supplementary Information

Title

Genotyping-by-sequencing based QTL mapping for rice grain yield under reproductive stage drought stress tolerance

Shailesh Yadav[1], Nitika Sandhu[1, 2], Vikas Kumar Singh[3], Margaret Catolos[1] and Arvind Kumar[1,4*]

[1] Rice Breeding Platform, International Rice Research Institute, DAPO Box 7777, Metro Manila, Philippines

[2] Punjab Agricultural University, Ludhiana, Punjab, India

[3] International Rice Research Institute, South Asia Hub, ICRISAT, Patancheru, Hyderabad, India

[4] IRRI South Asia Regional Centre (ISARC), Varanasi, Uttar Pradesh, 221106, India

[*]Author for Correspondence

Arvind Kumar
Director
IRRI South Asia Regional Centre (ISARC)
NSRTC Campus, G.T. Road, Collectry Farm
P.O. Industrial Estate, Varanasi-221006, Uttar Pradesh, India
Telephone: 0091-542-2518900
Email: a.kumar@irri.org
ORICID ID: 0000-0002-5488-9410

Figure S1: A comparative map showing narrowed down genomic region of previously identified *qDTY*$_{1.1}$ using high density linkage maps

a). *qDTY*$_{1.1}$ with undesirable linkages of early flowering and tallness (Vikram et al. 2011) located in genomic region of 36.75-40.70 Mb with QTL size of 3.95 Mb

b). *qDTY*$_{1.1}$ (present study) located nearly to 40 Mb genomic region with QTL size of 1.2 Mb size and free from undesirable linkages

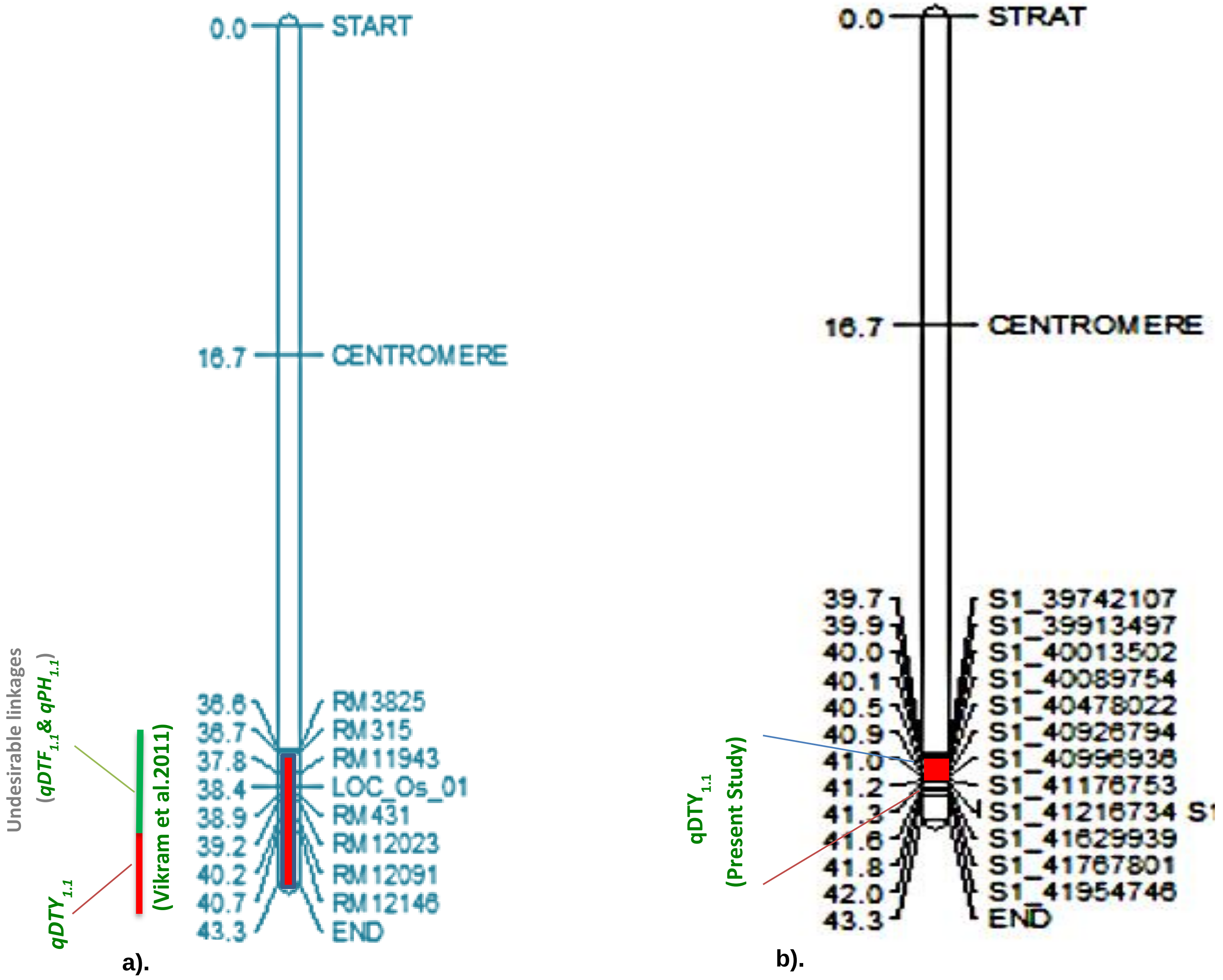

Supplementary Tables

Detailed Excel file has been attached (Table S1-S9)

Table S1. Summary of analyzed GBS data through TASSEL-GBS pipeline

Table S2. Alignment of reads to reference genome in Swarna*2/Dular population.

Table S3. Alignment of reads to reference genome in IR11N121*2/Aus 196.

Table S4. Total SNPs generated in TASSEL GBS pipeline for Swarna*2/Dular population.

Table S5. Total SNPs generated in TASSEL GBS pipeline for IR11N121*2/Aus 196 population.

Table S6. Filtered, polymorphic SNPs used in linkage map construction for Swarna*2/Dular population.

Table S7. Filtered, polymorphic SNPs used in linkage map construction for IR11N121*2/Aus 196 population.

Table S8. Validation of $qDTY_{1.1}$ using flanking markers in 250 lines randomly selected from five mapping populations

Table S9. Results of estimation of QTL effects among + QTL and - QTL lines
